# Performance Enhancement of Electrocatalytic Hydrogen Evolution through Coalescence-Induced Bubble Dynamics

**DOI:** 10.1021/jacs.4c02018

**Published:** 2024-03-27

**Authors:** Aleksandr Bashkatov, Sunghak Park, Çayan Demirkır, Jeffery A. Wood, Marc T. M. Koper, Detlef Lohse, Dominik Krug

**Affiliations:** † Physics of Fluids Group, Max Planck Center for Complex Fluid Dynamics and J. M. Burgers Centre for Fluid Dynamics, University of Twente, Enschede 7500 AE, Netherlands; ‡ Leiden Institute of Chemistry, Leiden University, Leiden 2333 CC, Netherlands; § Soft Matter, Fluidics and Interfaces, MESA+ Institute for Nanotechnology, J. M. Burgers Centre for Fluid Dynamics, University of Twente, Enschede 7500 AE, Netherlands; ∥ Max Planck Institute for Dynamics and Self-Organization, Göttingen 37077, Germany

## Abstract

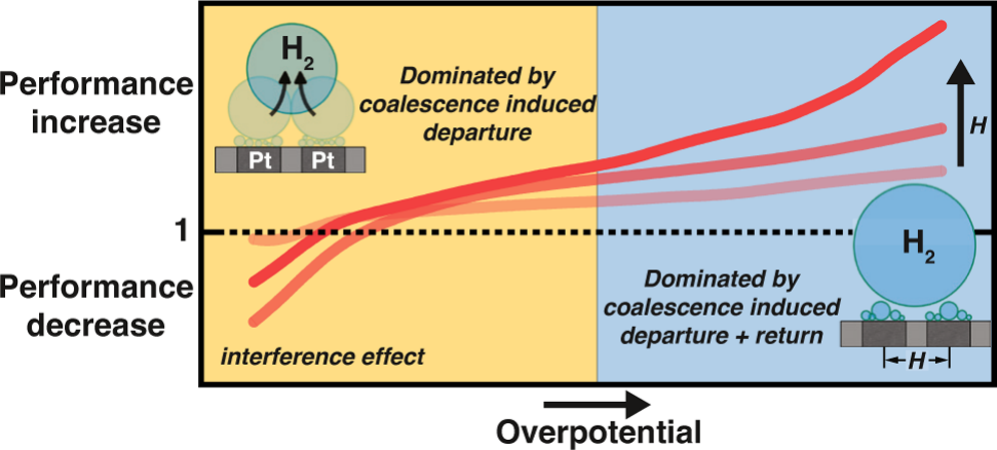

The evolution of electrogenerated gas bubbles during water electrolysis can significantly hamper the overall process efficiency. Promoting the departure of electrochemically generated bubbles during (water) electrolysis is therefore beneficial. For a single bubble, a departure from the electrode surface occurs when buoyancy wins over the downward-acting forces (e.g., contact, Marangoni, and electric forces). In this work, the dynamics of a pair of H_2_ bubbles produced during the hydrogen evolution reaction in 0.5 M H_2_SO_4_ using a dual platinum microelectrode system is systematically studied by varying the electrode distance and the cathodic potential. By combining high-speed imaging and electrochemical analysis, we demonstrate the importance of bubble–bubble interactions in the departure process. We show that bubble coalescence may lead to substantially earlier bubble departure as compared to buoyancy effects alone, resulting in considerably higher reaction rates at a constant potential. However, due to continued mass input and conservation of momentum, repeated coalescence events with bubbles close to the electrode may drive departed bubbles back to the surface beyond a critical current, which increases with the electrode spacing. The latter leads to the resumption of bubble growth near the electrode surface, followed by buoyancy-driven departure. While less favorable at small electrode spacing, this configuration proves to be very beneficial at larger separations, increasing the mean current up to 2.4 times compared to a single electrode under the conditions explored in this study.

## Introduction

Water electrolysis is likely to become a central technology in the CO_2_-neutral energy system of the future. Apart from being a potential energy carrier and fuel, hydrogen gas serves as a feedstock for the chemical (e.g., ammonia production for fertilizers) and steel industries (coal replacement) and refineries (methanol and synthetic fuels).^1−3^ Yet, the process efficiency requires further improvement to compete in the energy market and enable large-scale hydrogen production. In both conventional alkaline and proton exchange membrane water electrolyzers, a considerable part of the overpotentials and hence losses can be attributed to the formation of H_2_ and O_2_ bubbles present at the electrodes and in the bulk.^4−7^ These bubbles block the electrodes by masking their active surface area, reducing the number of nucleation sites. Additionally, they raise ohmic resistance by blocking the ion-conducting pathways.^8−10^ It is therefore vital to maintain a bubble-free electrode area for continuous catalytic activity. Enhanced removal of gas bubbles and deeper insights into their evolution processes will benefit further optimization of the system’s energy efficiency.^11^

Various methods have been developed to aid bubble departure, categorized as active (e.g., sonication, centrifugal forces, mechanical convection, pressure modulation, and external magnetic fields) and passive approaches.^5,7,12,13^ Passive methods, preferred for their energy efficiency, primarily involve surface modifications to alter the wettability^14^ of the catalytic surface.^15^ For example, superhydrophilic surfaces facilitate earlier bubble departure due to the reduced contact angle at liquid–solid interfaces.^16−22^

The bubble removal process can also benefit from the hydrophobic surfaces. One example is the bubble-free electrolysis concept that employs a hydrophobic porous layer adjacent to a porous electrode. This prevents bubble formation within the catalyst, guiding the generated gas by capillary effects through the hydrophobic layer.^23−26^ A different concept to enhance gas removal, which was shown to hold promise based on theoretical analysis,^27^ is the use of hydrophobic islands on the electrode as preferential nucleation sites. Also practically, this has been shown to be feasible using electrodes partially covered with hydrophobic spots made of polytetrafluoroethylene (PTFE).^28−30^ This allows us to guide the produced gas away from the active areas of the electrode with the potential to lower the bubble-induced overpotentials.^28,29^ Brussieux et al.^30^ demonstrated that, depending on the size of and distance between islets, parameters of the gas release, such as bubble size and location, can be controlled but did not study the effect on electrode performance. More recently, Lake et al.^31^ found that densely packed Pt-coated micropost arrays promote consistent release of smaller bubbles through their mutual coalescence. While this enhanced the stability of the current compared to untextured electrodes, it did not lead to performance gains when normalizing the active surface area in this system due to additional bubbles forming in between the pillars. In this context, the coalescence-induced removal of bubbles is of particular interest. Coalescence leads to a reduction in surface energy, and this difference is in part converted to kinetic energy, causing the bubble to jump off the surface without having to rely on buoyancy. This makes this removal process highly attractive in microgravity applications.^32−38^

However, a detailed understanding of the mechanism and quantification of the extent to which coalescence-induced dynamics can be exploited to improve the performance of gas-evolving electrodes is still lacking. This also applies to parameter optimization, which, in view of complications such as a possible bubble return to the electrode surface,^38−44^ is highly nontrivial. We address these open questions in the present work by studying the coalescence-driven dynamics of hydrogen bubbles produced at a dual microelectrode during water electrolysis. This new setup allows precise control of important parameters such as the bubble size during coalescence while also providing excellent observability of the dynamics. We demonstrate that coalescence events may lead to both premature bubble departure compared with buoyancy effects alone and the return of departed bubbles to the surface of the electrode, substantially altering the reaction rates. The dual microelectrode configuration shows, depending on the applied potential and interelectrode distance, up to a 2.4-fold increase in current compared to a single microelectrode.

## Experimental Section

**Figure 1:**
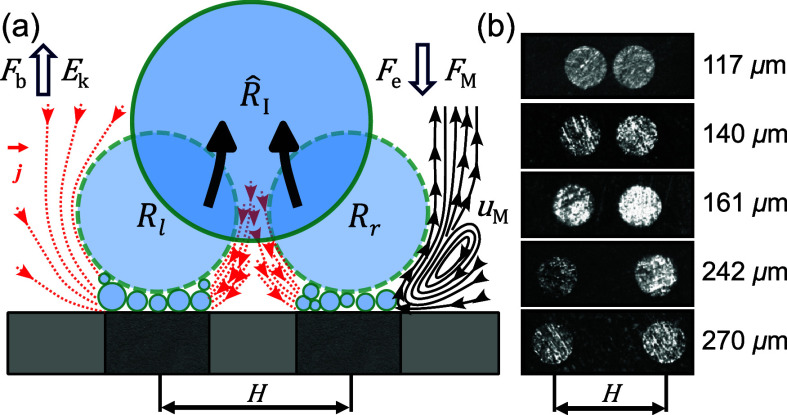
(a) Schematic of the dual microelectrode and two H_2_ bubbles sitting on the carpet of microbubbles. Each growing bubble is subject to a force balance including buoyancy, electric, and Marangoni forces. The red lines represent current density (*j*) and the black streamlines on the right represent the Marangoni convection with velocity *u*_M_. *E*_k_ is the kinetic energy released during the coalescence of the left (*R*_l_) and right (*R*_r_) bubbles. (b) Top view of the five dual microelectrodes with different interelectrode distance (*H*).

The pairs of H_2_ bubbles sketched in Figure 1a were generated at the surface of a dual platinum microelectrode during the hydrogen evolution reaction (HER). The experiment was performed in a three-electrode electrochemical cell filled with 0.5 M H_2_SO_4_ (for details see Supporting Information).

The fabrication of dual microelectrodes followed a previously established method.^45^ Briefly, two Pt wires (Ø100 μm, 99.99%, Goodfellow) were sealed into a soda-lime glass capillary (outer diameter Ø1.4 mm, inner diameter Ø1.12 mm, Hilgenberg) by gently softening the capillary in a flame. Five different values for interelectrode distance *H* were established and tested, as shown in Figure 1b. The electrode surface underwent electrochemical cleaning (potential cycling between 0.03...1.35 V vs RHE, repeated 50 times) after being mechanically polished with sandpaper (2000 grit), sonicated, and rinsed with ultrapure water. The cell used here closely resembles that used in earlier studies.^44−46^ The dual microelectrode (cathode) was inserted horizontally facing upward in the base of a cuboid glass cuvette (Hellma) with dimensions of 10 × 10 × 40 mm^3^. The system was completed by the reference electrode (Ag/AgCl) and a counter electrode (Ø 0.5 mm Pt wire) both inserted vertically from the top. The electrochemical cell was controlled by a potentiostat (BioLogic, VSP-300, 6 channels) at a constant potential of −0.2 to −2.8 V (vs RHE). Each of the two electrodes was connected to and controlled by a separate channel of the potentiostat. For each experimental run, the electric current was recorded with a sampling rate of at least 1 kHz over a period of 30 s. The optically transparent cell allows the visualization of the bubble dynamics using a shadowgraphy system. It consists of LED illumination (SCHOTT, KL 2500) with a microscope, connected to a high-speed camera (Photron, FASTCAM NOVA S16), providing a spatial resolution of 996 pix/mm. Image recording was typically performed at 5 kHz, unless otherwise stated. High-speed recording up to 264 kHz was used to resolve individual coalescence events. The bubble radius was extracted by standard image processing routine based on the Canny edge detection method in MATLAB R2022b (for further details see Supporting Information in Bashkatov et al.^47^). To measure the velocity fields around H_2_ bubbles presented in Figure 6, monodisperse polystyrene particles (microParticles GmbH) of Ø5 μm were seeded into the electrolyte. These particles are neutrally buoyant, with a mass density of 1.05 g/cm^3^. The resulting series of images, recorded at 1000 frames per second, were processed by the software DaViS 10, which employs a Particle Tracking Velocimetry (PTV) algorithm to track each particle over 25 ms shortly before departure. Due to the limited number of particles close to or at the bubble–electrolyte interface, the resulting tracks of the particles were collected for 60 bubbles. Subsequently, the tracks were converted into a vector field using a binning function that interpolates local tracks on a specified fine grid. The videos are available in the Supporting Information.

## Results and Discussion

### Single Electrode

**Figure 2:**
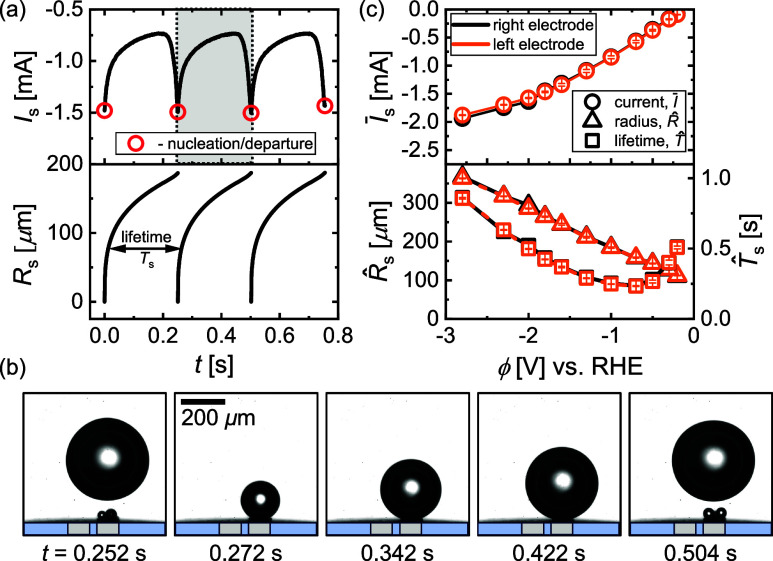
(a) Electric current and radius over time representing three complete cycles of bubble evolution at ϕ = −1.0 V at a single microelectrode. The red circles mark the nucleation and departure instants of time. (b) Shadowgraphs displaying the evolution cycle, marked in gray in (a). (c) Average electric current (circles), departure radius (triangles), and lifetime (squares) versus the potential for the right (black) and left (orange) electrodes run separately. Image recording performed at 500 frames/second.

To set the baseline, we briefly report the results for the case where only a single electrode is operated, which has been studied previously.^38,44,46−55^ As an example, Figure 2a shows the transient current (*I*_s_) and radius (*R*_s_) of the bubble for three complete bubble evolution cycles at −1.0 V. Shadowgraphs corresponding to a complete cycle from nucleation^56−58^ to departure are included in Figure 2b. This process is highly periodic with a bubble lifetime of *T*_s_. The evolution of the bubble has a strong influence on the reaction current, for which the maxima in cathodic current marked by the red circles coincide with the departure of the bubble. This is immediately followed by the nucleation of a new bubble, whose growth in the vicinity of the electrode leads to a considerable reduction in *I*_s_ of up to 50% in this case. This continues until the next bubble departure, after which the cycle repeats itself.

Finally, Figure 2c summarizes how the average electric current , where the overline denotes an average over *t*, the departure radius , and lifetime  varies for different cathodic potentials (ϕ). All statistics are averaged over multiple bubble cycles, with error bars representing the standard deviation. The figure also confirms that consistent results are obtained from both electrodes.

In this system, bubble formation occurs already at low overpotentials. Micron-sized bubbles form on the electrode surface and continuously coalesce to form a single larger bubble. This larger bubble is typically not in direct contact with the electrode surface but rather resides on the layer of microbubbles.^44^ It continues to grow via intensive coalescence with these microbubbles and via gas diffusion.^59^ In this case, departure of the bubble occurs once the retaining forces due to the electric field,^44,60,61^ thermal,^62−65^ and solutal^45,66,67^ Marangoni effects are overcome by buoyancy (see Figure 1a). The thermal Marangoni effect is related to the Joule heating caused by the locally high current density (*j*) at the bubble foot, as indicated in Figure 1a. The effect therefore scales (via Ohm’s law) with *j*^2^ and prevails at high overpotentials. The solutal effect due to the depletion of the electrolyte at the electrode is expected to depend linearly on *j* and therefore dominates at lower overpotentials (ϕ ≳ −0.7 V in the present case).^45^ The electric force is directly proportional to ϕ and therefore all retaining forces diminish as the overpotential is reduced, which explains the decreasing trend of the departure radius  as |ϕ| is made less negative. Since the bubble captures almost all the produced gas,^45,46^ the departure period follows from the time it takes to produce the gas contained in the bubble volume and *T*_s_ is therefore proportional to *R*_s_^3^/*I*.

### Dual Electrode

#### Modes of Bubble Evolution

**Figure 3:**
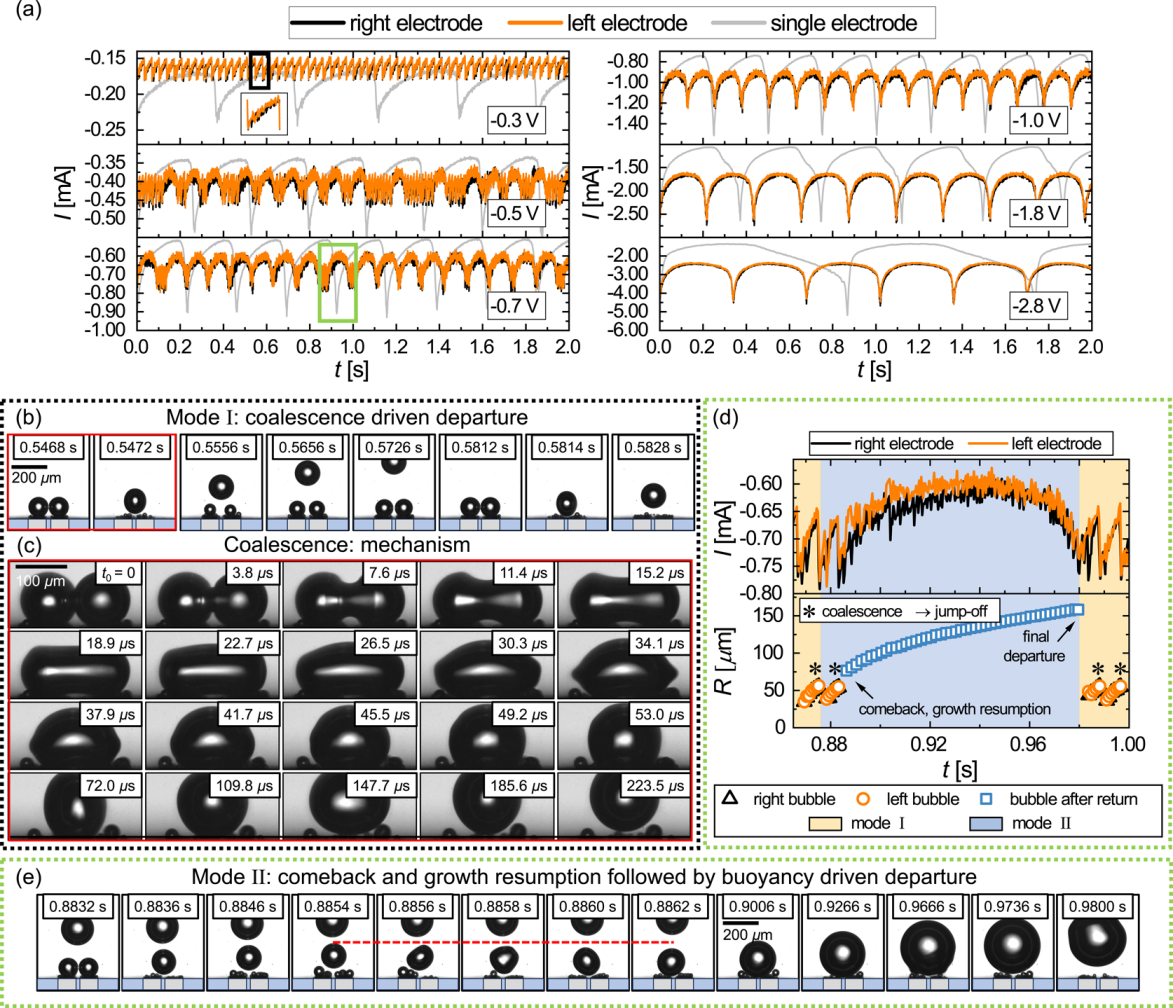
(a) Electric current over 2 s (out of 30 s) of the experimental run at various potentials (ϕ). The black and orange curves represent the electric current measured at the right and left electrodes, respectively. Gray lines represent the corresponding results for single electrode operation. (b) Snapshots depict the bubble evolution following mode I as indicated in (a) by the black rectangular inset at −0.3 V. (c) Snapshots detailing the coalescence-driven departure process recorded at −0.5 V. *t*_0_ is one frame before the coalescence process begins. (d) Zoomed-in view of the current at −0.7 V, shown by the green rectangle in (a), with corresponding evolution of *R*(*t*). The orange and blue shades correspond to modes I and II, respectively. (e) Mode II of bubble evolution from (d). The red line indicates the maximum height reached by the departed bubble. Recordings in (b,e) were performed at 5 kHz and at 264 kHz in (c).

From now on, both electrodes are operated simultaneously, independently of each other and at the same potential. Initially, we will only consider the pair with a separation of *H* = 117 μm. The measured currents for this configuration are plotted in Figure 3a for different potentials. Time traces of the current for both electrodes (“left” and “right”) are included, and for reference, we also show the current signal measured when only a single electrode is operated at the same potential (gray line). Focusing initially on the lowest overpotential, ϕ = −0.3 V, the current oscillations remain periodic during dual operation; however, both the period and amplitude are notably diminished. The reason for this can be understood from the corresponding shadowgraphs presented in Figure 3b, which illustrate the bubble dynamics over one period (shown by a black box in Figure 3a).

Similar to what is observed for a single electrode, a larger bubble forms and grows initially at each of the two electrodes, leading to a gradual decrease in the current. This process continues until the two bubbles touch and coalesce, which is followed by the departure of the merged bubble along with a spike in the current (see inset at −0.3 V in Figure 3a). Figure 3c details this coalescence process, which happens on the order of microseconds, and the emerging deformations of the bubble shape. The coalescence-induced jump-off is powered by the released surface energy.^38,68−70^ While the majority of this energy is dissipated through the capillary waves seen in Figure 3c,^37,71^ the fraction that is transformed into kinetic energy (less than 1%, for details see Supporting Information) can cause bubble departure at much smaller radii than in the buoyancy-driven scenario, for the newly formed bubble. Together with the fact that each of the coalescing bubbles contributes only half of the volume, this explains the significantly enhanced departure frequency.

At higher overpotentials at ϕ = −0.5 V, events with a much longer period length start appearing intermittently in the current traces. These events become more frequent and dominate the signal at ϕ = −0.7 V, before almost fully superseding the high-frequency coalescence pattern at ϕ ≤ −1.0 V. In order to elucidate the underlying bubble dynamics, we provide an enlarged view of a segment of the current signal at ϕ = −0.7 V (green box) in Figure 3d along with the size evolution of the bubbles. The first bubble departure included in Figure 3d proceeds analogously to the one shown in Figure 3b, and the bubble continues to rise away from the electrode after the coalescence-induced takeoff. We will refer to this as “mode I” from now on. However, as the corresponding shadowgraphs in Figure 3e show, even though the bubble also jumps off after the second coalescence event, it is eventually brought back to the surface through repeated coalescence with newly formed bubbles at both electrodes (see the period between *t* = 0.8854 s and *t* = 0.8862 s). Following this return, the bubble rests between the two electrodes just above the surface. There, it continues to grow until a buoyancy-driven departure (at *R*_II_ = 158 μm vs *R*_I_ = 72 μm), which explains an order of magnitude longer lifetime (*T*_II_ = 104.4 ms vs *T*_I_ = 8.4 ms) of the bubble in this instance. We will denote this as “comeback mode” or “mode II”.

**Figure 4:**
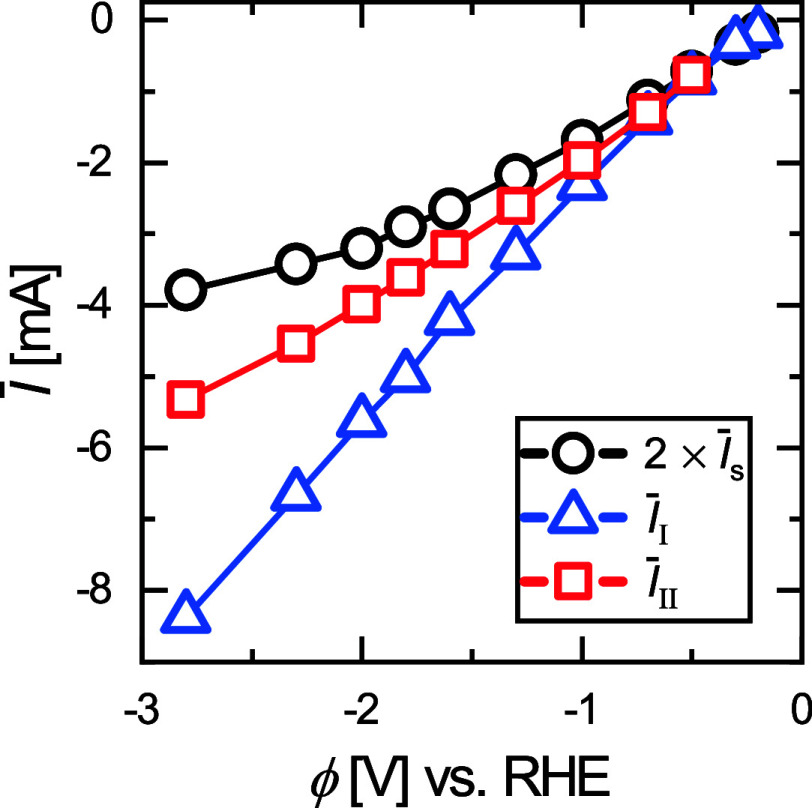
Electric current  vs potential (ϕ) for the single electrode (black) and for modes I (blue) and II (red) at dual microelectrode. Both  and  are the sum of the currents at the left and right electrodes.

It is evident from Figure 3a that the dynamics induced by coalescence have a strong impact not only on the current fluctuations but also on the mean current at a specific potential. To analyze this, we compare period averaged currents for the two modes ( and , taken to be the sum of the currents at both electrodes) to  in Figure 4. Note that it is possible to determine *I*_I_ even at high potentials where mode II prevails by considering only the time until the first coalescence, leading to a temporary departure of the bubble (see Figure 3d). Despite the much faster gas removal, the current at low overpotentials (ϕ ≳ −0.7 V) remains the same or even slightly decreases in dual operation compared to the single electrode case. This can be attributed to the additional shielding by the second bubble in the vicinity of the electrode and the diffusive competition between the two reaction sites, both of which lower the performance. However, the benefits of accelerated gas removal increasingly outweigh these effects as the overpotential is increased. This is particularly true for mode I, where the current is more than double that of the single electrode at the same potential for the most negative values of ϕ investigated. While this clearly demonstrates the potential for performance enhancement through coalescence-induced gas removal, the effective performance enhancement is reduced to less than 50% for the current electrode spacing due to the prevalence of bubble return (mode II) at higher overpotentials. The currents in mode II are consistently lower compared to mode I because the electrode separation is so small that the returning bubble still blocks a large part of both electrodes (see Figure 3e), even though it is located halfway between them.

#### Phase Diagram

**Figure 5:**
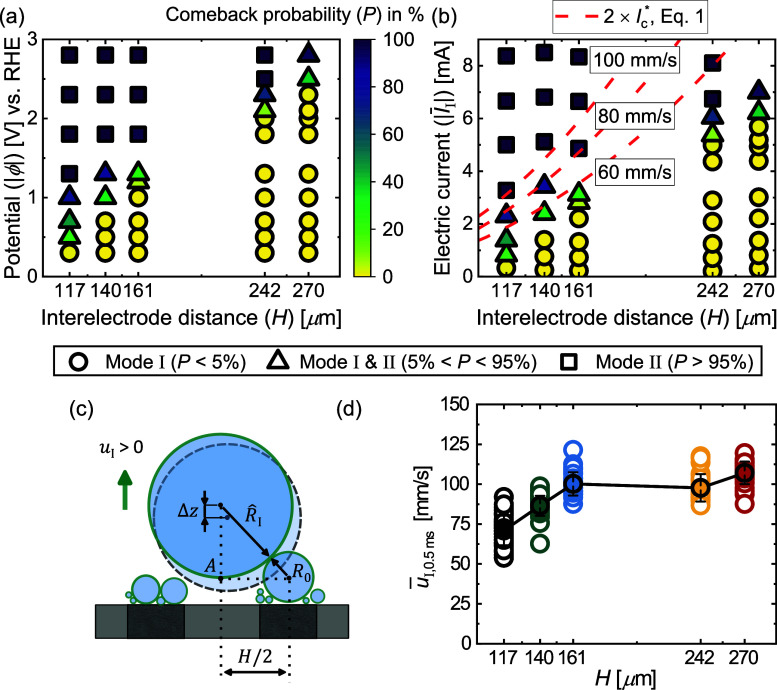
Phase diagram representing the probability (*P*) of the bubble coming back after initial departure shown in terms of (a) potential and (b) current vs *H*. The color bar scales the probability from 0 to 100%. The circles denote Scenario I, i.e., when *P* is less than 5%, and squares denote Scenario II, with *P* more than 95%. The triangles are for a mixed regime, where the probability varies widely from 5 to 95%. The red lines plot 2 × *I*_c_* using eq 1. (c) Sketch illustrating the relevant geometry for the bubble return. (d) Vertical jump velocity  averaged over the first 0.5 ms of the jump vs *H* for numerous bubbles. The line represents the averaged values at each *H*, completed with the standard deviation.

**Figure 6:**
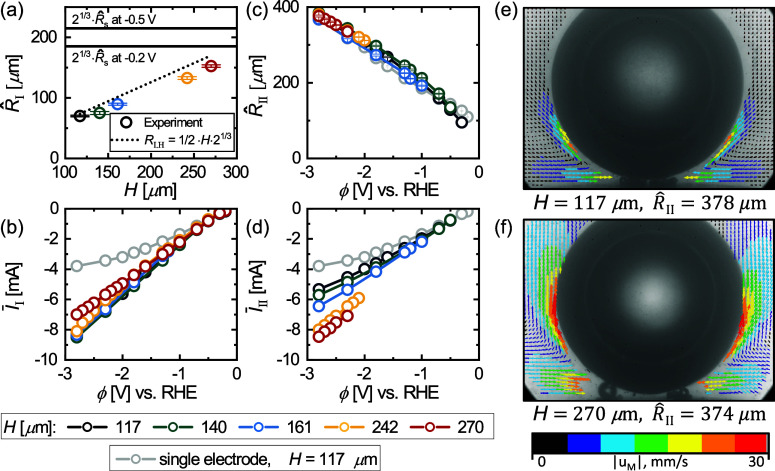
Departure radius (a) , (c)  and electric current (b) , (d)  for modes I and II, respectively.  is given as a function of *H*. , , and  are shown as functions of potential and for various *H*. Gray curves are for the single electrode. (e,f) Velocity fields, |*u*_M_|, representing Marangoni convection during mode II at −2.8 V and *H* = 117 μm and *H* = 270 μm, respectively. The velocity is measured in a period of 25 ms before the bubble departure.

To better understand under what conditions the return of the bubble after jump-off happens, Figure 5 documents the probability (*P*) of return for different interelectrode distances (*H*) and as a function of ϕ (Figure 5a) and  (Figure 5b). As *H* is increased, the transition from mode I (*P* < 5%, circles), to a mixed regime (5% ≤ *P* ≤ 95%, triangles), and finally to mode II (*P* > 95%, squares) occurs at increasingly larger values of |ϕ|. In fact, the dependence on *H* is quite strong: for a fixed potential of ϕ = −1.3 V, *P* changes from about 100% at *H* = 117 μm to almost 0 when the distance is increased to *H* = 270 μm. The sketch in Figure 5c illustrates the relevant mechanism for bubble return. A newly formed bubble (with radius *R*_0_) on one of the electrodes catches up and coalesces with the departed bubble with radius . Due to momentum conservation, the resulting bubble is then located at the joint center of mass of the two coalescing bubbles, which implies a downward shift by Δ*z* compared to the location of the bubble with radius . Repeated coalescence events from both sides then bring the bubble back to the surface, as seen in Figure 3e. The transition between mode I and mode II is therefore governed by a competition between the departure or “jump” velocity of a bubble after coalescence and the growth rate of bubbles at the electrode. A larger magnitude of electric current, increasing approximately linearly with ϕ (see Figure 6), enables faster formation of new bubbles, which then increases the likelihood of their interaction with the previously departed bubble. Upon increasing *H*, the bubble-successor needs to grow to a larger size, hence for a longer time before interacting with the already-departed bubble, allowing the latter to move farther away. This will dramatically increase the current required for the comeback mode. We can capture this in a simple model based on the geometry sketched in Figure 5c to predict the minimum current *I*_c_ for bubble return. Our analysis considers the situation where the new bubble with radius *R*_0_ has grown large enough to get in contact with the departing bubble. The time it takes for the bubble to grow to the radius *R*_0_ is Δ*t* = *kR*_0_^3^/*I*_c_, where  is a prefactor containing the Faraday constant *F*, the pressure inside the bubble *P*_g_, the gas constant *R*_g_, and the temperature *T* (see Supporting Information for details). During this time interval, the departing bubble travels the distance Δ*t*·*u*_I_, with *u*_I_ denoting the effective jump velocity. Based on the geometry of the triangle spanned by the centers of the two bubbles and the point A in Figure 5c, the critical current for the mode transition as a function of *R*_0_ is given by
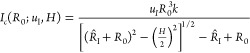
1

For any *H*, a value of *R*_0_ can be determined for which *I*_c_ reaches a minimum value, *I*_c_*. To obtain the value of the current *I*_c_* in this critical configuration, an estimate of the jump velocity is required. To obtain this, we tracked bubbles departing after coalescence and then averaged their vertical velocity over the first 0.5 ms to obtain . Note that *u*_I_ varies widely depending on the position of both bubbles before coalescence (see Supporting Information for details). The results for this quantity are shown in Figure 5d as a function of *H*. From these data, typical values for *u*_I_ are found to be in the range from 60 to 110 mm/s with a slight tendency toward higher velocities as the bubble size increases at larger electrode separations *H*. In Figure 5b, we have included results for 2 × *I*_c_* as a function of *H* and for different values of *u*_I_. It can be seen that the model very well captures the increase in the critical current as the electrode separation increases. The best agreement between the model and the data is for *u*_I_ = 60 mm/s, which is close to, although slightly lower, than the measured jump velocities in Figure 5d. Among potentially other factors, a reason for this slight difference is the fact that the new bubble with radius *R*_0_ is also formed by coalescence and therefore also jumps off the electrode. Additionally, we do not account for the shape oscillations of the larger bubbles, which become more prevalent at larger *H*.

#### Performance vs Interelectrode Distance, *H*

To understand how the current varies at different electrode separations, it is useful to first consider how the departure size of the bubbles changes for different *H*. In mode I, the departure is coalescence-driven so that  is independent of ϕ and varies only with the interelectrode distance *H*. Due to lateral oscillations of the bubble position on the electrode and possibly a slight inclination of the electrode surfaces, the results for  shown in Figure 6a are about 10% lower than 2^–2/3^*H*, i.e., the value for the coalescence of two bubbles each with a radius of *H*/2. This small difference was taken into account when evaluating  in eq 1. Once *H* exceeds two times the departure radius at the single electrode (2 × *R*_s_), each of the two bubbles will depart due to buoyancy before coalescence happens. The maximum radius *R*_I_ is therefore given by , and this limit is indicated in Figure 6a by black solid lines for the electrode with *H* = 270 μm at −0.2 and −0.5 V (see Figure S3 in Supporting Information for relevant reference data).

Compared to the single electrode, the current in mode I shown in Figure 6b is most enhanced at high overpotential and small *H* because, in this case, the reduction in bubble departure size is maximal. There is only a moderate decrease in  for larger *H*, primarily due to the relatively small range in *H* and, consequently, in , which is minor compared to variations observed in  at different potentials. At low overpotentials,  for the larger electrode separations studied, and there is no increase in the current compared to *I*_s_, just as was observed at *H* = 117 μm in Figure 4.

In mode II, the departure radius strongly depends on the potential but at most weakly on *H*, as shown in Figure 6c. Remarkably,  is approximately the same as for the single electrode case at the same potential (see gray symbols representing ). An investigation of the force balance^72−74^ leading to these trends in  is beyond the scope of this study. Nevertheless, we present clear evidence of Marangoni convection (see Figure 6e,f), consistent with the presence of thermocapillary effects in the same potential range on single electrodes.^51,65^ Based on the flow direction, a resulting downward Marangoni force on the bubble is expected (see Figure 1a). The convective motion is much more pronounced at *H* = 270 μm (Figure 6f) compared to the narrower spacing of *H* = 117 μm in Figure 6e, which is in line with the difference in current between the two cases ( vs 8.46 mA, respectively). Interestingly, this does not result in a noticeable difference in  for the different interelectrode distances, which is presumably due to differences in the geometry-dependent electric force.^74^ We confirmed that the continued coalescence with small bubbles does not exert a significant apparent force on the bubble (see Supporting Information for details).

In contrast to mode I, the current in mode II shown in Figure 6d shows a clear dependence on the electrode separation and increases strongly for a larger *H*. This is because the bubble is now centered in between the two electrodes. Therefore, the electrodes become more exposed as the distance between them increases, even if the bubble size remains the same. The continuous removal of the smaller bubbles on the electrode by coalescence with the larger one proves to be very beneficial and leads to maximum currents of more than twice , equaling the largest currents observed in mode I.

**Figure 7:**
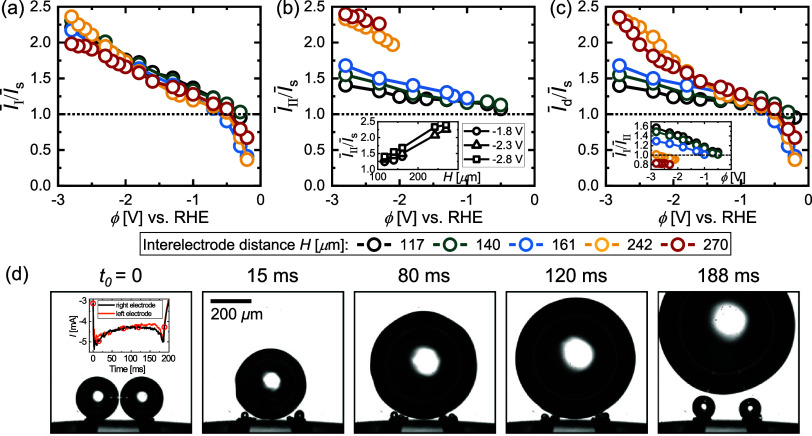
Electric current (a) , (b) , and (c) , all in dimensionless form with reference to . Data are presented as a function of potential (ϕ) and interelectrode distance (*H*). The inset in (b) shows  vs *H* at −1.8, −2.3, and −2.8 V. The inset in (c) documents  vs ϕ.  is the current averaged over both mode I (*I*_I_) and mode II (*I*_II_). (d) Snapshots throughout the bubble evolution at −2.8 V and *H* = 270 μm. *t*_0_ = 0 marks an instant of time one image before the coalescence of two bubbles (with radii *R*_l_ and *R*_r_, respectively), followed by the jump of the merged bubble off the electrode and its consecutive return. The inset shows the electric current throughout the entire evolution, with the red circles marking the corresponding snapshots.

To quantify the performance gain and to compensate for the ϕ dependence of the current, we normalize the current on the dual electrode by . This also accounts for small differences in  between the different electrodes used in this study (see Supporting Information). In Figure 7a, the ratio  is plotted for different *H* as a function of ϕ. As the figure shows, the interference effects at low overpotentials, already discussed in the context of Figure 3, cause  to even fall below  for ϕ ≳ −0.5 V. This does not improve noticeably for larger electrode spacing, presumably due to a trade-off between reduced interference effects and the increase in the bubble size with *H*. For larger overpotentials, the benefits of the enhanced gas removal prevail, reflected in a ratio  which also consistently increases with increased overpotential exceeding a value of 2 at ϕ = −2.8 V. Approximately the same values are also encountered for this potential for the ratio  in Figure 7b. While the performance in mode II also improves slightly for a higher overpotential, it most strongly depends on *H*. As the inset in Figure 7b shows, the ratio  increases approximately linearly with *H* at constant potential.

Finally, Figure 7c shows how the resulting effective current on the dual electrode  changes relative to . In addition to variations in *I*_I_ and *I*_II_, this quantity is also influenced by the probability *P*(*H*, ϕ) of the bubble return (mode II). Given the results in Figure 5a, the ratio  is therefore dominated by mode I at low and by mode II at large overpotentials. This implies that the performance gains in mode I at high |ϕ| are not practically realizable. However, this is only a limitation at smaller electrode separations, since the current in mode II even exceeds that of mode I for *H* = 242 μm and *H* = 270 μm (see the inset of Figure 7c). For these cases, the mode transition is therefore even beneficial.

Figure 7d shows snapshots for the parameter combination *H* = 270 μm and ϕ = −2.8 V for which the highest ratio  was observed. Having the returned bubble located at the center between the electrodes avoids the formation of larger bubbles directly on the electrodes. Notably, only a slight drop in the current is observed (see inset at *t*_0_ = 0) as the outline of the bubble moves beyond the electrode positions. This contradicts the common practice of considering the region under the bubble as inactive but is in line with earlier conjectures.^31,75^

### Conclusions

We have explored the coalescence dynamics of electrogenerated bubbles and their influence on the electrochemical reaction rate using dual platinum microelectrodes. We found that the coalescence of two adjacent bubbles leads to an initial jump-off of the merged bubble and premature escape from the surface. However, the continued coalescence with newly formed successors may result in a return to the electrode and hence prolonged growth. The latter mode is increasingly prevalent when the current is higher and the interelectrode distance is smaller. We proposed a simple model to capture these trends and predict the critical magnitude of the current required to initiate the return process. It is noteworthy that gravity only plays a secondary role in the coalescence dynamics, and the buoyancy effect is not included in the model. Therefore, we also expect similar dynamics on a vertical electrode, with minor modifications caused by the asymmetry introduced in this configuration. This comeback mode negates the potential performance improvement achieved through direct departure following the coalescence event at a smaller *H* (up to a 1.7- vs 2.3-fold increase in current at constant potential when compared to a single electrode). However, even in cases of bubble return, the effective current at larger *H* increased by up to 2.4 times because the bubble was then located between the electrodes, exposing a greater electrode area for the reaction. Therefore, this mode is promising, especially since, given the dependence on electrode separation, even greater performance gains can be expected by further increasing *H*. However, once *H* exceeds two times the departure radius at the single electrode (2 × *R*_s_), each of the two bubbles will depart due to buoyancy before coalescence happens. In such a case, similar to what is observed at lower potentials (see Figure 7a, below ca. |0.5| V), the resulting current is expected to be lower than that at a single electrode. In practice, a similar configuration may be achieved on extended electrodes using hydrophobic islands, which should be spaced to favor coalescence-based departure and minimize the probability of bubble return, thus avoiding the blocking of the active surface area. Albeit not studied here, we expect that our results will generally also apply to, for example, the oxygen evolution reaction or to different electrolytes. However, one should account for differences, e.g., due to a different gas production rate at the same current and potential changes to the coalescence efficiency based on the bubble departure size, when designing electrodes.

## Supporting Information Available

Single electrode, −1.0 V, Figure 2b (MP4)
Mode I dual electrode, −0.3 V, Figure 3b (MP4)
Coalescence-departure mechanism, −0.7 V, *H* 270 μm (MP4)
Mode II dual electrode, −0.7 V, Figure 3e (MP4)
Mode II dual electrode, coalescence-comeback mechanism, −1.3 V, *H* 117 μm (MP4)
Marangoni convection, −2.8 V, *H* 117 μm, Figure 6e (MP4)
Marangoni convection, −2.8 V, *H* 270 μm, Figure 6f (MP4)
Mode II dual electrode comparison, −2.8 V, *H* 117 μm, Figure 7 (MP4)
Mode II dual electrode comparison, −2.8 V, *H* 270 μm, Figure 7 (MP4)
Experimental setup; single electrode: characterization; dual electrode: characterization; phase diagram: model; and apparent force: bubble–carpet interaction (PDF)
