# Supplementary material for: Performance Enhancement of Electrocatalytic Hydrogen Evolution through Coalescence-Induced Bubble Dynamics
Source: J Am Chem Soc. 2024 Mar 27;146(14):10177–86. doi: 10.1021/jacs.4c02018 (PMC11009962; doi:10.1021/jacs.4c02018)
Supplement: Supplementary file 10 — ja4c02018_si_010.pdf [file ja4c02018_si_010.pdf]

Supporting Information: Performance enhancement of electrocatalytic hydrogen evolution through coalescence-induced bubble dynamics

Aleksandr Bashkatov,[*,†] Sunghak Park,[‡] Çayan Demirkır,[†] Jeffery A. Wood,[¶] Marc T.M. Koper,[‡] Detlef Lohse,[†,§] and Dominik Krug[*,†]

†*Physics of Fluids Group, Max Planck Center for Complex Fluid Dynamics and J. M. Burgers Centre for Fluid Dynamics, University of Twente, Enschede, 7500 AE, Netherlands*

‡*Leiden Institute of Chemistry, Leiden University, Leiden, 2333 CC, Netherlands*

¶*Soft Matter, Fluidics and Interfaces, MESA+ Institute for Nanotechnology, J. M. Burgers Centre for Fluid Dynamics, University of Twente, Enschede, 7500 AE, Netherlands*

§*Max Planck Institute for Dynamics and Self-Organization, Göttingen, 37077, Germany*

E-mail: a.bashkatov@utwente.nl; d.j.krug@utwente.nl

Experimental setup

The pairs of H_2 bubbles were generated at the surface of a dual platinum micro-electrode during the hydrogen evolution reaction (HER). The experiments were performed in a three-electrode electrochemical cell filled with 0.5 M H_2SO_4, see figure S1a. The cell used here closely resembles that used in earlier studies.[1–3]

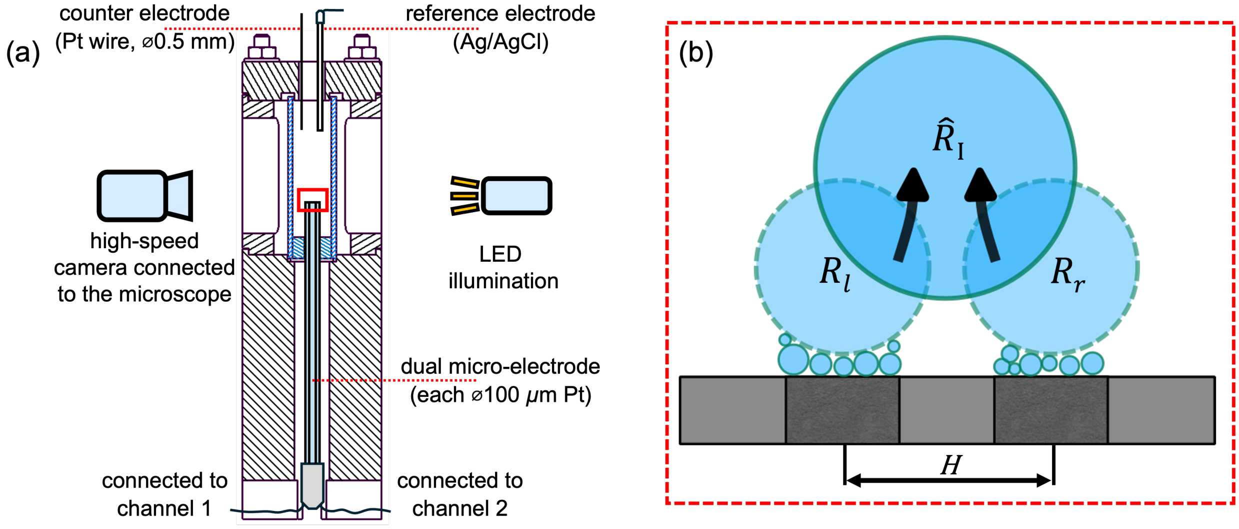

Figure S1: The schematics of (a) the electrochemical cell and (b) dual Pt micro-electrode.

The dual micro-electrode (cathode) is inserted horizontally facing upward in the base of a cuboid glass cuvette (Hellma) with dimensions of 10 × 10 × 40 mm^3. The dual micro-electrode consists of two Pt wires (∅100 μm, 99.99%, Goodfellow) sealed into a soda-lime glass capillary (outer diameter ∅1.4 mm, inner diameter ∅1.12 mm, Hilgenberg). The system is completed by the reference electrode (Ag/AgCl) and counter electrode (∅ 0.5 mm Pt wire) both inserted vertically from the top. The electrochemical cell is controlled by a potentiostat (BioLogic, VSP-300, 6 channels) at a constant potential of -0.2 to -2.8 V (vs. RHE). Each of the two electrodes is connected to and controlled by a separate channel of the potentiostat. The optically transparent cell allows visualization of the bubble dynamics

using a high-speed shadowgraphy system. It consists of LED illumination (SCHOTT, KL 2500) with a microscope, connected to a high-speed camera (Photron, FASTCAM NOVA S16), providing a spatial resolution of 996 pix/mm. To measure the velocity fields around H_2 bubbles, monodisperse polystyrene particles (microParticles GmbH) of ∅5 μm were seeded into the electrolyte. These particles are neutrally buoyant with a mass density of 1.05 g/cm^3.

The combination of a micro-electrode and sulfuric acid makes it possible to produce single hydrogen bubbles at each individual electrode. In figure S1b, a schematic depicts a pair of bubbles slightly above the dual micro-electrode, sitting on and continuously coalescing with a carpet of microbubbles. Typically, these bubbles continue to grow until they reach a critical radius $R_l = R_r$, which is solely determined by the interelectrode distance H. At this point, they come into close contact and eventually coalesce. The resulting bubble, with a radius of $\hat{R}_I$, will jump off of the electrode surface.

Single electrode: characterization

Figure S2 documents the electric current over 5 seconds (out of 30 seconds) of the experimental run at various potentials (ϕ). The currents are shown for $H = 117$ μm for left and right electrodes (see figure 1a in the manuscript), run separately.

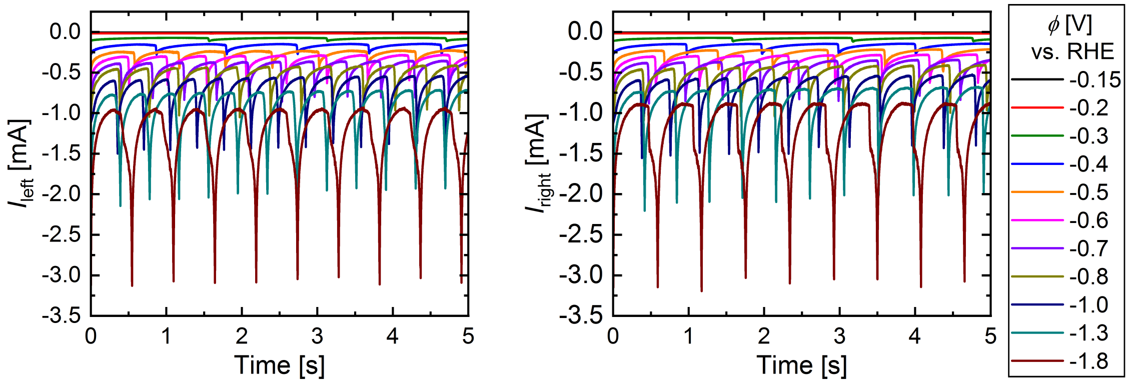

Figure S2: The electric current over time for left and right electrodes.

Figure S3 characterizes five electrodes in terms of the electric current ($2 \times \overline{I}_s$), lifetime ($\hat{T}_s$), and radius at the departure ($\hat{R}_s$) vs. ϕ. $\overline{I}_s$ is averaged over 30 seconds and the left

and right electrodes. $\hat{T}_s$ and $\hat{R}_s$ are averaged for multiple bubbles and accompanied by the standard deviations. The low standard deviation for $\hat{R}_s$ and $\hat{T}_s$ demonstrates the highly

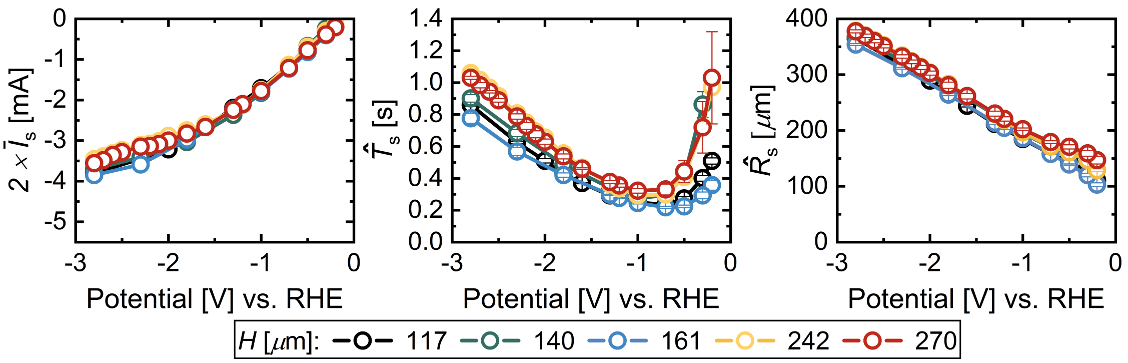

Figure S3: The electric current, lifetime and departure radius for H_2 bubbles produced at single electrode vs. ϕ for different electrodes (H). The error bars represent standard deviation.

periodical evolution of bubbles. The quite similar current between various H suggests that the surfaces of these electrodes are alike. However, the small differences are enough to affect the dynamics of H_2 bubbles and significantly alter the lifetime and size at the departure.

Dual electrode: characterization

For completeness of the results presented in the manuscript figures S4 and S5 document the electric current (I) vs. time plotted for 1 second our of 30 seconds of the experiment run. I is plotted for various potentials (ϕ) and interelectrode distance (H). Figs. S4(a), (b) and (c) are for $H = 117\,\mu$m, $H = 140\,\mu$m, $H = 161\,\mu$m, respectively. Figs. S5(a) and (b) are for $H = 242\,\mu$m and $H = 270\,\mu$m.

Figure S6 documents the lifetime of the bubbles produced at dual electrode vs. potential (ϕ) for Modes I (left) and II (right) and for different electrodes. $\hat{T}$ is averaged for multiple bubbles and accompanied by the standard deviations. Two main trends can be observed: (i) Since the departure radius in mode I is independent of the ϕ, $\hat{T}_I$ reduces at larger overpotentials, owing to higher electric current. It also increases together with H, especially at larger ϕ. — This is because the pair of bubbles need to grow to a bigger size before coalescence at

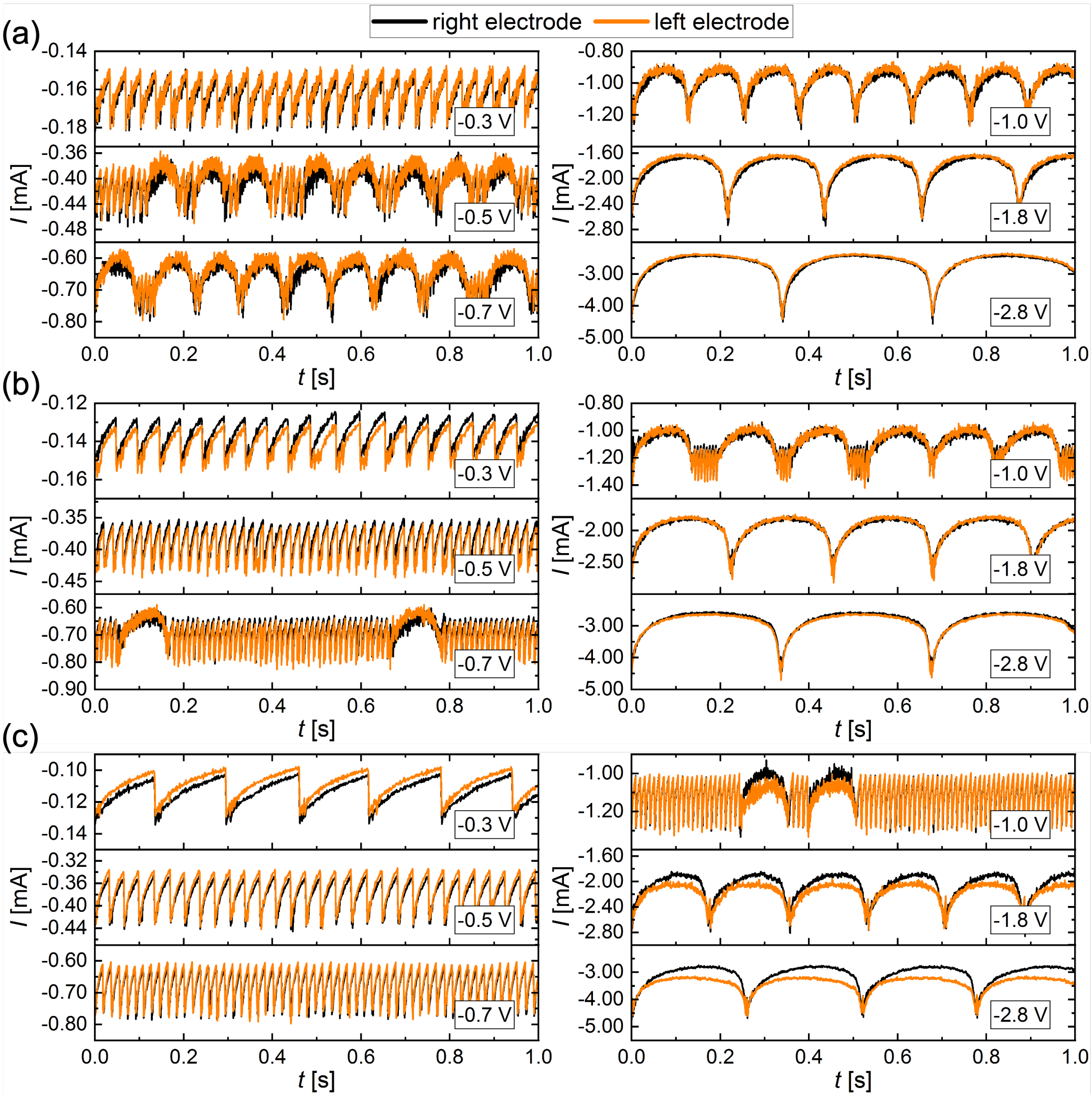

Figure S4: The electric current vs. time plotted for 1 second out of 30 seconds of the experimental run for various potentials ϕ and interelectrode distance: (a) $H = 117\mu$m, (b) $H = 140\mu$m, (c) $H = 161\mu$m.

larger H; (ii) On the other hand, $\hat{T}_{II}$ increases at larger overpotentials and reduces at larger H. As already mentioned in the manuscript, larger overpotentials imply larger downward-acting forces increasing the departure size of the bubble. Therefore the bubble would grow for a longer time. However, the separation of two electrodes away from each other for larger distances (H) enables higher currents, hence faster H_2 production rate.

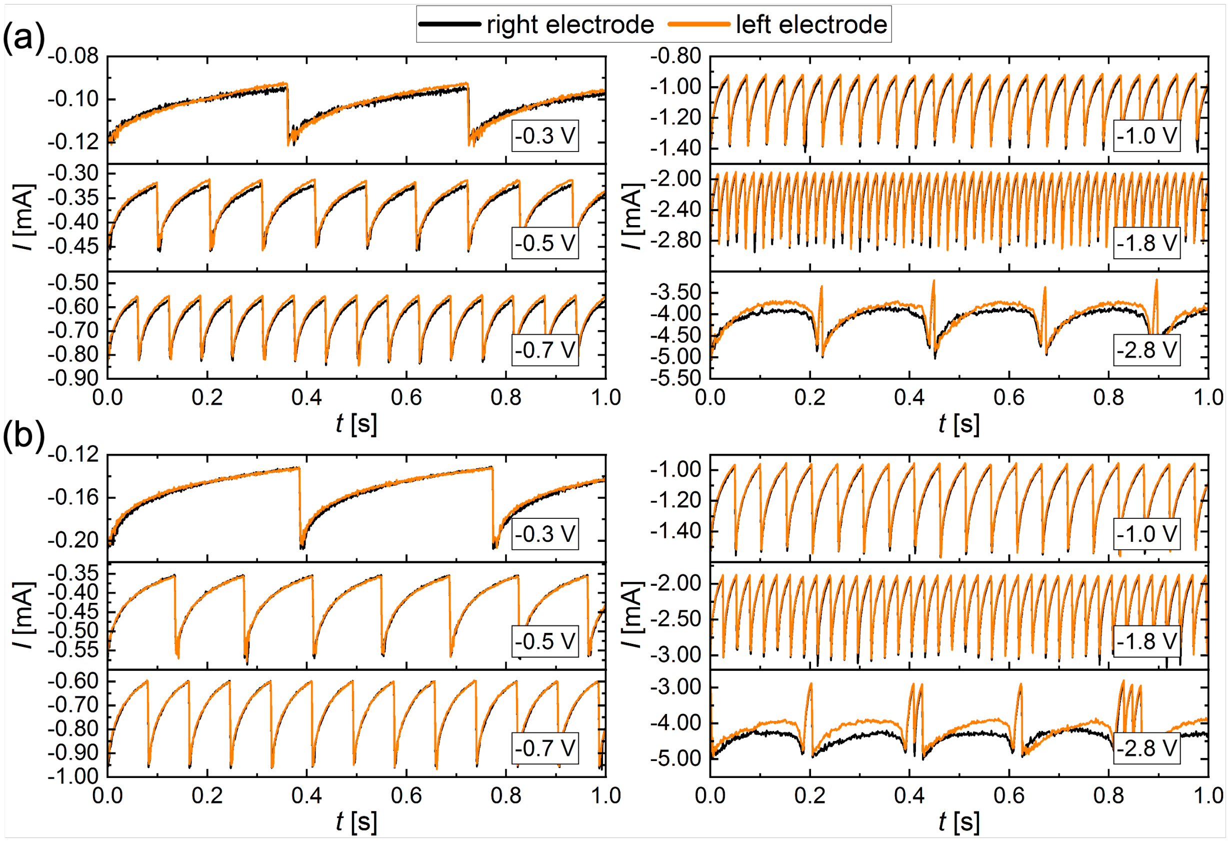

Figure S5: The electric current vs. time plotted for 1 second out of 30 seconds of the experimental run for various potentials ϕ and interelectrode distance: (a) $H = 242$ μm, (b) $H = 270$ μm.

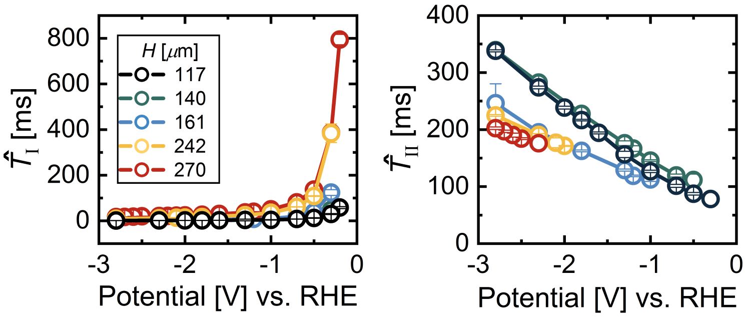

Figure S6: The lifetime at dual electrode as a function of potential (ϕ) and interelectrode distance (H). $\hat{T}_I$ and $\hat{T}_{II}$ are for mode I and mode II, respectively. The error bars represent standard deviation.

Figure S7 demonstrates the traveling distance in vertical direction of the merged bubble in the first 5 milliseconds after the jump-off of the electrode driven by the coalescence event at $H = 117$ μm. The results are shown for $\phi = -0.3$ and -0.5 V and three bubbles in each case. The curves document that the jumping velocity notably decays over time (Δt) due to the effects of drag force, particularly in the moments following the jump. Interestingly,

the "terminal" velocity of the bubble, which can be read from the slope of the curves and i.e. ca. after 2 ms, increased at a larger overpotential (-0.5 V). This might be explained by the wake behind the rising bubble-predecessor. This flow drags, hence accelerates the merged bubble in the moment of departure. The wake enhances with the faster departure of bubbles-predecessors (smaller T) which is the case at a more negative potential.

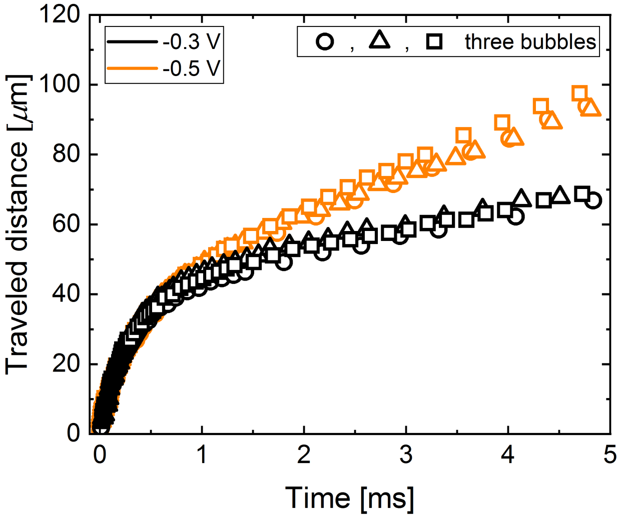

Figure S7: The trajectory of bubble at -0.3 V and -0.5 V over the first 5 milliseconds after coalescence driven jump-off. $t_0 = 0$ is one frame before coalescence. Each potential is presented by three bubbles. The interelectrode distance $H = 117$ μm.

Figure S8 represents the vertical jumping velocity $\overline{u}_{I.0.5\,ms}$ for numerous bubbles, averaged over the first 0.5 ms of the jump, vs. parent size ratio R_s/R_l. The experiments performed at $H = 117$ and (a) $\phi = -0.5$ V, (b) $\phi = -1.0$ V. R_s and R_l are radii for smaller and larger bubbles, respectively. The geometric parameters are shown in figure S8c. The color bar scales another geometric parameter Y_{max} given in dimensionless form. It represents the relative position of the bubble i.e. the distance from the bubble bottom to the electrode, chosen as the maximum value between the smaller and bigger bubbles. The bubble sits at the electrode if $Y = 1$ and is away from the electrode if $Y > 1$. The circles represent mode I, i.e. when the bubble departs into the bulk following the coalescence event, and squares denote mode II, i.e. when the once departed bubble following the coalescence event comes back to the surface, continues to grow, and departs at a later stage due to buoyancy. Note

that u_I varies widely depending on the position of both bubbles before coalescence and their size ratio.

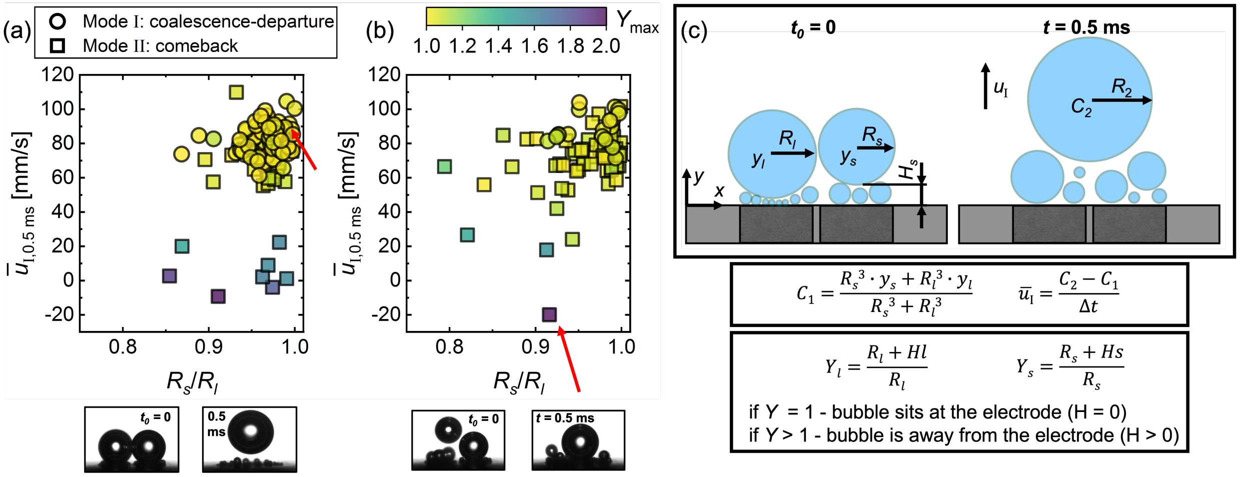

Figure S8: (a),(b) The vertical jumping velocity ($\overline{u}_{I,0.5ms}$) vs.. R_s/R_l at $\phi = -0.5$ V and $\phi = -1.0$ V. The color bar scales the relative position of the bubble shown in (c), i.e. the maximum of either smaller or bigger bubbles, prior to coalescence at $H = 117$. Image recording performed at 10 kHz.

Figure S8(a) and (b) demonstrates two general trends: (i) $\overline{u}_{I,0.5ms}$ reduces as per reduction in R_s/R_l and as per increase in Y_{max}; (ii) at higher potential, hence current, the bubble would come back to the electrode more often moving away with even higher jumping velocity, as already emphasized in the manuscript (see figure 5).

Upon coalescence of two bubbles, there is a release of surface energy (ΔG_s) given as

$$\Delta G_s = 4\pi\gamma(R_l^2 + R_r^2 - R_I^2), \tag{1}$$

where R_l, R_r and R_I are the left, right and merged bubbles, respectively. $\gamma \approx 0.072$ N/m is the surface tension of the electrolyte. The released energy partly dissipates by the bubble oscillations, working against viscous drag. When in the proximity to the surface, the remaining energy is converted to the kinetic energy (E_k) driving the resultant (merged) bubble to jump off the electrode.[4] The kinetic energy is

$$E_k = \frac{1}{2}C_M\rho_l\frac{4}{3}\pi R_I^3 u_I^2, \tag{2}$$

where C_M is added mass coefficient, ρ_l electrolyte density and u_I is the initial jumping velocity. For a spherical bubble $C_M = 0.5$, however, when the bubble is in proximity to the wall the coefficient is larger.[5] In detail, when two bubbles approach each other, the thin film of electrolyte separating them gradually drains $\mathcal{O}(\mu\text{s})$ and eventually ruptures. This leads to the formation of a neck, i.e. an open cavity, and a series of capillary waves of varying strengths that propagate along the electrolyte-gas interface. These waves move away from the neck region until they meet at the opposite apex of the coalescence point (see manuscript, fig. 3c). The strength of these waves decreases as they travel along the interface due to continuous viscous dissipation.[6] Meanwhile, the surface tension γ drives the retraction of the remaining capillary waves towards a spherical shape, deforming the bubble shape. Once the excess surface energy overcomes the work done by the bubble against viscous drag (W_μ) during the expansion and retraction processes, the resultant net component of momentum perpendicular to the surface causes the bubble to jump off the electrode. As neither of the bubbles is attached to the electrode, the adhesion energy W_a is neglected. The process is controlled by surface tension and viscosity and is often characterized in terms of the Ohnesorge number ($Oh = \frac{\mu}{\sqrt{\rho \gamma R_I}}$).[6] μ is the dynamic viscosity of the electrolyte. The influence of gravity during the coalescence, before lift-off, is negligible.[5] While the process is considered highly inefficient, with only a small portion of surface energy translating into kinetic energy,[5] it is sufficient for a resultant bubble (R_I) to jump off the electrode acquiring an initial velocity u_I.

Figure S9 documents an estimation of the ratio between the translated kinetic energy (E_k) and the released surface energy (ΔG_s) as a function of (a) R_I and (b) H. The data points in fig. S9a is calculated using eq. 1 and eq. 2 by taking corresponding R_l, R_r, R_I and $\overline{u}_{I,0.5ms}$ for the bubbles presented in fig. S8a, i.e. at $H = 117\,\mu$m and $\phi = -0.5$ V. The circles represent the bubbles following mode I and squares mode II. The data points in fig. S9b is based on the $\hat{R}_I$ from fig. 6a and $\overline{u}_{I,0.5ms}$ from fig. 5d in the manuscript, assuming that $R_l = R_r$, hence $R_l = \frac{\hat{R}_I}{2^{1/3}}$.

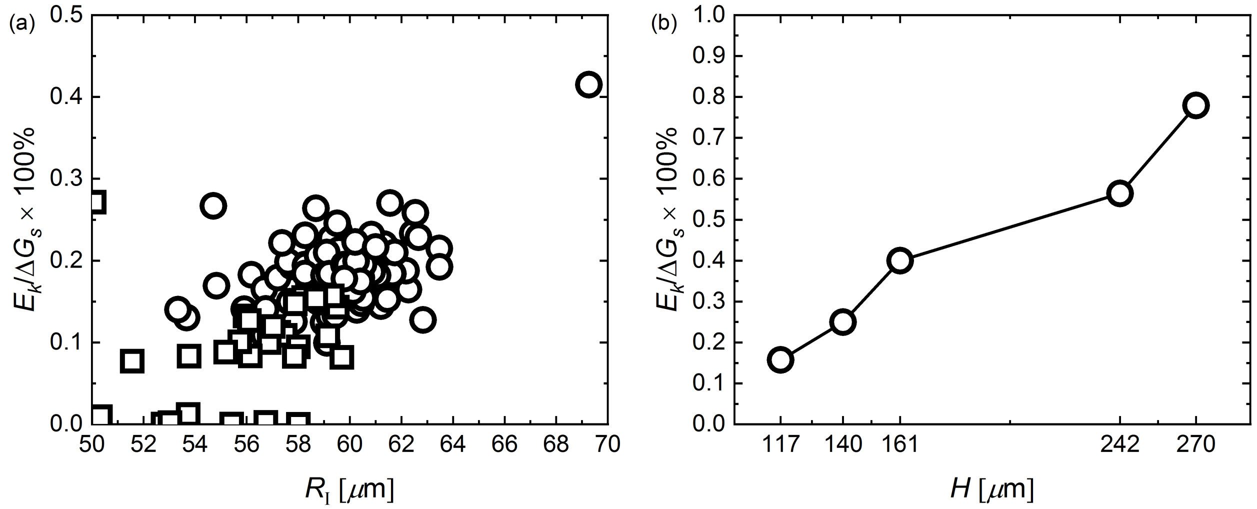

Figure S9: The ratio between the translated kinetic energy (E_k) and the released surface energy (ΔG_s) as a function of (a) R_I and (b) H. The line in (b) is to guide the eye.

Although the taken velocity $\overline{u}_{I,0.5ms}$ is smaller than the initial jumping velocity and is further affected by the wake behind the departed bubble-predecessor, fig. S9 confirms the inefficiency of the coalescence process, as the translated kinetic energy is below 1% of the released surface energy.

Phase diagram: model

To provide a general understanding of the relationship between probability (P), interelectrode distance (H), and electric current (I) presented in fig. 5 (see manuscript), we propose a simple model based on the velocity of the departing bubble and the growth rate of the bubble-successor. The model predicts the critical magnitude of the current $\overline{I}_c$ required to produce hydrogen quickly enough to guarantee the coalescence between the departing bubble ($\hat{R}_I$) and its successor (R_0). The time it takes for the new bubble to grow to the radius R_0 is Δt which can be found from Faraday's and ideal gas laws.

The ideal gas law given as

$$P_g V = \frac{m}{M} R_g T, \tag{3}$$

where P_g is the pressure inside the bubble, V is the volume of the gas, m is the total mass of the gas, M is the molar mass, R_g is the ideal gas constant and T is temperature of the gas. The produced charge is

$$Q = \overline{I}_c \cdot \Delta t. \tag{4}$$

The Faraday's law reads

$$\frac{m}{Q} = \frac{1}{F}\frac{M}{\nu}, \tag{5}$$

where F is the Faraday's constant and ν is the valency of the ions (+1) multiplied by number of protons (2).

By substituting Eqs. 4 and Eq. 5 in Eq. 3

$$\Delta t = \frac{R_0^3}{\overline{I}_c} \cdot \frac{8\pi}{3}\frac{FP}{R_g T} = \frac{R_0^3}{I_c} \cdot k, \tag{6}$$

with $k = \frac{8\pi}{3}\frac{FP}{R_g T}$. During this time interval Δt, the departing bubble travels the distance $\Delta t \cdot u_I$, with u_I denoting the effective jump velocity. Based on the geometry of the triangle spanned by the centers of the two bubbles and the point A in figure 5c (see manuscript), the following relation can be expressed:

$$\left[(\hat{R}_I + u_I \cdot \Delta t) - R_0\right]^2 + \left(\frac{H}{2}\right)^2 = (\hat{R}_I + R_0)^2 \tag{7}$$

where $\hat{R}_I = 2^{1/3} \cdot \frac{H}{2}$ or is taken from experiment (see figure 6a).

By substituting Δt into eq. 7, the critical current for the mode transition as a function of R_0 is given by:

$$I_c(R_0; u_I, H) = \frac{u_I R_0^3 k}{\left[(\hat{R}_I + R_0)^2 - \left(\frac{H}{2}\right)^2\right]^{1/2} - \hat{R}_I + R_0}. \tag{8}$$

Apparent force: bubble-carpet interaction

It was shown that the H_2 bubble grows while seated on a carpet of microbubbles, undergoing a continuous intensive coalescence with it. Throughout numerous coalescence events, the mother bubble experiences a shift in the mass center, resulting in acceleration towards the electrode surface. This acceleration can be considered as an apparent force F_{g-g}. An estimate for this apparent force is derived from the principle of momentum conservation, given as

$$mc_M = m_0 c_0 + \dot{m} t c, \tag{9}$$

where m and c_m represent the total mass and mass center of the bubble at time t, respectively; m_0 and c_0 denote the initial mass and mass center of the bubble at an arbitrary time $t_0 = 0$; and $\dot{m}$ stands for the mass injection rate due to continuous coalescence with the carpet of microbubbles with the center of mass at c. m_0 and c_0 are constant. All mass centers are with reference to the electrode surface. The mass of the bubble m at any given moment is

$$m = m_0 + \dot{m} t. \tag{10}$$

By substituting $\dot{m} t = m - m_0$ from eq. 9 into eq. 10

$$mc_M = m_0 c_0 + mc - m_0 c. \tag{11}$$

Given that c at a given current I is a constant (as well as m_0 and c_0)

$$m\dot{c_M} = \dot{m}(c - c_M). \tag{12}$$

We further assume that $\dot{m}$ is also constant at given I. By performing differentiation one more time using eq. 12

$$m\ddot{c_M} = -2\dot{m}\dot{c_M}. \tag{13}$$

By substituting eq. 12 in eq. 13

$$F_{g-g} = m\ddot{c_M} = -2\frac{\dot{m}}{m}\dot{m}(c - c_M). \tag{14}$$

When considering only a short interval t, we can approximate $c_M \approx R$ and eq. 14 reads

$$F_{g-g} = 2\frac{1}{m}(\frac{m}{t})^2 R. \tag{15}$$

c, which is the thickness of the carpet, is also neglected. Using the Faraday's and ideal gas laws

$$\dot{m} = \frac{m}{t} = \frac{\rho_g V}{V} = \frac{\rho_g Itk}{t} = \rho_g Ik, \tag{16}$$

where $k = \frac{R_g T}{2P_g F}$.

Finally, by substituting eq. 16 into eq. 15 the apparent force reads

$$F_{g-g} = \frac{3}{2}\frac{\rho_g I^2 k^2}{\pi R^2}. \tag{17}$$

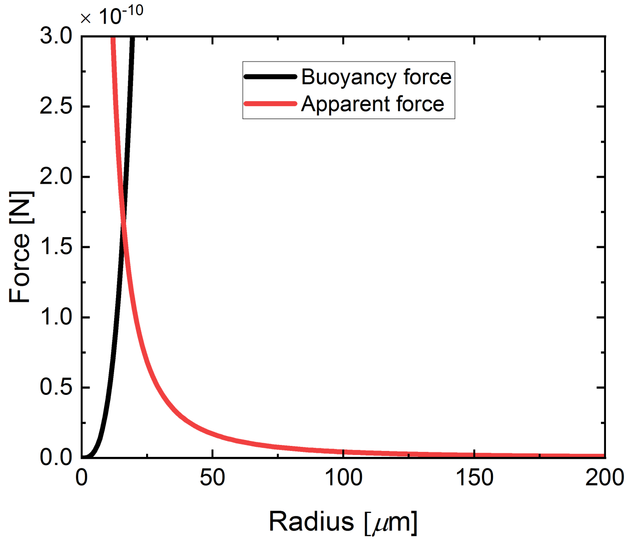

Figure S10: Buoyancy and apparent forces as a function of bubble radius at $I = -8$ mA.

Figure S10 documents a comparison between the buoyancy force and apparent force,

calculated using eq. 17 at $I = -8$ mA (which corresponds to approximately the maximum current observed in this study), against the bubble radius R. The buoyancy force defined as $F_b = (\rho_g - \rho_{H2})gV$, where g represents gravitational acceleration. Fig. S10 demonstrates that the continued coalescence with the carpet of microbubbles does not impose a significant apparent force on the bubble. — This force decays rapidly with increasing R and becomes smaller than the buoyancy force at at approximately $R = 16\,\mu$m.

References

(1) Yang, X.; Karnbach, F.; Uhlemann, M.; Odenbach, S.; Eckert, K. Dynamics of single hydrogen bubbles at a platinum microelectrode. *Langmuir* **2015**, *31*, 8184–8193.

(2) Bashkatov, A.; Hossain, S. S.; Mutschke, G.; Yang, X.; Rox, H.; Weidinger, I. M.; Eckert, K. On the growth regimes of hydrogen bubbles at microelectrodes. *Physical Chemistry Chemical Physics* **2022**, *24*, 26738–26752.

(3) Park, S.; Liu, L.; Demirkır, Ç.; van der Heijden, O.; Lohse, D.; Krug, D.; Koper, M. T. Solutal Marangoni effect determines bubble dynamics during electrocatalytic hydrogen evolution. *Nature Chemistry* **2023**, 1–9.

(4) Lv, P.; Peñas, P.; Eijkel, J.; Van Den Berg, A.; Zhang, X.; Lohse, D.; others Self-propelled detachment upon coalescence of surface bubbles. *Physical Review Letters* **2021**, *127*, 235501.

(5) Raza, M. Q.; Köckritz, M. v.; Sebilleau, J.; Colin, C.; Zupancic, M.; Bucci, M.; Troha, T.; Golobic, I. Coalescence-induced jumping of bubbles in shear flow in microgravity. *Physics of Fluids* **2023**, *35*.

(6) Sanjay, V.; Lohse, D.; Jalaal, M. Bursting bubble in a viscoplastic medium. *Journal of Fluid Mechanics* **2021**, *922*, A2.
